# Thoracoabdominal Normothermic Regional Perfusion and Donation After Circulatory Death Lung Use

**DOI:** 10.1001/jamanetworkopen.2024.60033

**Published:** 2025-02-17

**Authors:** Isaac S. Alderete, Arya Pontula, Samantha E. Halpern, Kunal J. Patel, Jacob A. Klapper, Matthew G. Hartwig

**Affiliations:** 1 Duke University School of Medicine, Durham, North Carolina; 2 University of Manchester Medical School, Manchester, United Kingdom; 3 Department of Surgery, Massachusetts General Hospital, Boston; 4 Division of Cardiovascular and Thoracic Surgery, Department of Surgery, Duke University, Durham, North Carolina

## Abstract

**Importance:**

Donation after circulatory death (DCD) heart procurement has increased, but concerns remain about the effect of simultaneous heart and lung procurement, particularly with thoracoabdominal normothermic regional perfusion (TA-NRP), on the use of DCD lungs. Previous analyses exclude critical donor factors and organ nonuse, and rapidly rising DCD use may bias comparisons to historical controls.

**Objective:**

To use validated risk-adjusted models to assess whether DCD heart procurement via TA-NRP and direct procurement is associated with lung use.

**Design, Setting, and Participants:**

This retrospective cohort study involved adult DCD donors between January 1, 2019, and September 30, 2024, listed in the Scientific Registry of Transplant Recipients (SRTR). The SRTR deceased donor yield model was used to develop an observed to expected (O:E) yield ratio of lung use obtained through DCD among 4 cohorts: cardiac DCD donors vs noncardiac DCD donors and cardiac DCD donors undergoing TA-NRP vs direct procurement. Temporal trends in O:E ratios were analyzed with the Cochran-Armitage test.

**Main Outcomes and Measures:**

The O:E ratios of DCD lung use.

**Results:**

Among 24 431 DCD donors (15 878 [65.0%] male; median [IQR] age, 49.0 [37.0-58.0] years), 22 607 were noncardiac DCD (14 375 [63.6%] male; median [IQR] age, 51.0 [39.0-58.0] years) and 1824 were cardiac DCD (1503 [82.4%] male; median [IQR] age, 32.0 [26.0-38.0] years) donors; noncardiac DCD donors were more likely to be smokers (6873 [30.4%] vs 227 [12.4%]; *P* < .001). Among cardiac DCD donors, 325 underwent TA-NRP, while 712 underwent direct procurement. TA-NRP donors had shorter median (IQR) lung ischemic times (6.07 [4.38-9.56] hours vs 8.12 [6.16-12.00] hours; *P* < .001) and distances to recipient hospitals (222 [9-626] nautical miles vs 331 [159-521] nautical miles; *P* = .050) than direct procurement donors. Lung use was higher among cardiac DCD donations compared with noncardiac DCD donations (16.7% vs 4.4%, *P* < .001). Within the cardiac DCD cohort, lung use was similar between TA-NRP and direct procurement (19.1% vs 18.7%; *P* = .88) cohorts. Both noncardiac DCD and cardiac DCD donors had observed lung yields greater than expected (O:E, 1.29 [95% CI, 1.21-1.35] and 1.79 [95% CI, 1.62-1.96]; both *P* < .001), although cardiac DCD yield was significantly higher than noncardiac DCD yield (*P* < .001). Both TA-NRP and direct procurement lung yields were greater than expected (O:E, 2.00 [95% CI, 1.60-2.43] and 1.77 [95% CI, 1.52-1.99]; both *P* < .001) but were not significantly different from each other (*P* = .83). The O:E ratios did not change significantly over time across all cohorts. Among recipients, the TA-NRP cohort experienced significantly better 90-day mortality (0 of 62 vs 9 of 128 patients [7.0%]; *P* = .03) and overall survival (4 of 62 patients [6.5%] vs 21 of 128 patients [16.4%]; *P* = .04) rates compared with the direct procurement cohort.

**Conclusions and Relevance:**

In this cohort study of DCD donors, concomitant heart procurement provided better-than-expected rates of lung use as assessed with validated O:E use ratios regardless of procurement technique. The findings also suggest a survival benefit with improved 90-day and overall survival rates for the TA-NRP cohort compared with the direct procurement cohort. Policies should be developed to maximize the benefits of these donations.

## Key Points

**Question:**

Is simultaneous heart procurement, particularly with thoracoabdominal normothermic regional perfusion (TA-NRP), associated with the use of lungs procured through donation after circulatory death (DCD)?

**Findings:**

This cohort study of 24 431 DCD donors found significantly higher lung use among 1824 DCD donors who also donated the heart compared with 22 067 DCD donors who did not, with both groups exceeding expected lung yields. Among DCD heart donors, lung use rates were comparable between 325 TA-NRP donors and 712 direct procurement donors.

**Meaning:**

These results suggest that concomitant heart and lung procurement during DCD organ donation is associated with better-than-expected lung use regardless of procurement technique and that transplant policies should optimize use of these donations.

## Teaser

This cohort study uses a risk-adjusted analysis of the observed to expected ratios of lung use in the Scientific Registry of Transplant Recipients to investigate whether lung vs simultaneous heart and lung donation after circulatory death, and thoracoabdominal normothermic regional perfusion vs direct procurement, are associated with the use of donor lungs.

## Introduction

Donation after circulatory death (DCD) heart transplantation has increased in recent years and offers an important opportunity to expand the donor pool for cardiac transplantation.^1^ Growth in DCD heart transplantation can be partially attributed to improved procurement techniques broadly classified as thoracoabdominal normothermic regional perfusion (TA-NRP) or direct procurement, followed by either static cold storage or ex vivo machine perfusion.^2,3^ In both approaches, expeditious heart procurement is prioritized over lung procurement, as the heart is more vulnerable to ischemic injury. The organ procurement organization (OPO) and transplant center decide jointly to perform TA-NRP vs direct procurement prior to organ procurement. Prior work from members of our group^4^ suggests that compared with direct procurement, TA-NRP may be associated with suboptimal lung allograft function in the immediate postoperative period but similar graft survival at 1 year. Despite these data supporting conscientious use of DCD lung allografts after TA-NRP, the effect of concomitant heart procurement on overall DCD lung use rates remains unclear. Rates of DCD lung use have been increasing annually, making comparison with historical controls biased. Therefore, contemporaneous and controlled comparison is necessary.

Several aspects of concomitant heart procurement may injure lungs from DCD donors, potentially discouraging otherwise viable allografts. First, quick entry to the thoracic cavity alongside DCD donor coagulopathy may cause excessive bleeding, necessitating high-volume transfusion, which is associated with recipient primary graft dysfunction and mortality.^5,6^ Second, while use of venoarterial extracorporeal membrane oxygenation (VA-ECMO) has been associated with left ventricular dilation and pulmonary congestion in other settings, this use remains a theoretical concern with TA-NRP, in which VA-ECMO is sometimes used.^7^ Furthermore, variations in procuring team experience with TA-NRP may contribute to differences in lung quality and the decision to use the lungs for transplantation. Despite these concerns, several studies in the US have demonstrated comparable rates of DCD lung use with use of TA-NRP and direct procurement.^4,8,9^ However, these studies are limited by small cohorts and have not adjusted for donor risk factors, highlighting the need for larger, risk-adjusted analyses to fully address this question.

To better characterize the association of simultaneous heart procurement and TA-NRP with DCD lung use, we conducted a risk-adjusted analysis using a national cohort of DCD donors. Specifically, we identified controlled DCD donors in the United Network for Organ Sharing (UNOS) database and examined the observed to expected (O:E) ratios of lung use among 4 groups: noncardiac DCD donors, cardiac DCD donors, and, via subgroup analysis, cardiac DCDs procured with TA-NRP vs direct procurement. We hypothesized that cardiac donation is adversely associated with lung DCD use and that TA-NRP is associated with lower DCD lung use compared with direct procurement.

## Methods

### Data Source

We performed a retrospective cohort study using the UNOS Scientific Registry of Transplant Recipients (SRTR) database, which includes all organ donors in the US since October 1, 1987. The reporting of results adheres to the guidelines set out in the Strengthening the Reporting of Observational Studies in Epidemiology (STROBE) reporting guideline. This study was approved by the Institutional Review Board at Duke University, which deemed the study not human participants research, and thus the requirement for obtaining informed consent was waived.

### Study Population

All controlled DCD donors were considered for inclusion. Donors before January 1, 2019, were excluded, as the first DCD heart transplant in the US occurred in 2019. Data through September 30, 2024, were collected. We excluded non-DCD donors, donors under the age of 18 years, and donors with unclear lung disposition.

### Study Design

The primary objective of this analysis was to evaluate the association of DCD heart procurement with lung use for transplantation. The cohort was stratified into donors from whom a heart was recovered (cardiac donor), regardless of whether the heart was transplanted, and donors from whom a heart was not recovered (noncardiac donor). Rates of DCD lung use were compared between cardiac DCD and noncardiac DCD groups.

The second objective of this study was to evaluate the association of the DCD heart procurement technique (TA-NRP or direct procurement) with lung use. As procurement technique is not discretely captured in the dataset, the interval from time of death to time of thoracic aorta cold flush was used to define procurement technique. Within the cardiac DCD cohort, donors with a time interval of greater than 15 minutes were defined as TA-NRP; those with elapsed time less than 15 minutes were defined as direct procurement, as previously described.^4^ Donors with missing aorta clamp time or time of death were excluded from further analysis. Unadjusted rates of DCD lung use were compared between TA-NRP and direct procurement groups.

### Estimating Expected Organ Yield

The SRTR releases a risk-adjusted estimate of expected donor yield twice annually that assesses the likelihood of successful transplantation for a specific donor’s organs. Using logistic regression modeling, risk-adjusted estimates of organ-specific donor yield are developed using the most current data collected by the Organ Procurement and Transplantation Network (OPTN).^10^ These models have been validated and used in the literature to calculate expected yield of organs transplanted but, to our knowledge, have not been applied in lung transplantation.^11,12,13^ Once a model is chosen for a specific organ, the organ-specific expected yield is calculated algebraically using the model coefficients publicly shared by the SRTR and the OPTN UNOS database. In this way, lung use from each donor is adjusted for donor characteristics, allowing for comparison between observed and expected rates of lung use. For this analysis, we used the most recent model released in January 2024. The observed yield was divided by the expected yield. A ratio of 1 indicated equal observed yield and expected yield; ratios greater or less than 1 suggested that observed yield exceeded or fell short of the expected yield, respectively.

We sought to determine whether the observed organ yield in each group was statistically different from the expected yield and from the observed yield in the comparator groups. To do so, we computed the O:E ratio as observed yield divided by expected yield. Bootstrapping techniques were employed to develop 95% CIs and 2-sided *P* values around the O:E ratios under a null hypothesis positing that observed yield was exactly equal to expected yield (ie, O:E ratio of 1.0).^14^ We used 1000 bootstrapped samples for all cohorts.

### Subanalysis

The TA-NRP use among OPOs was also characterized to better understand variations in practice. Trends in TA-NRP use and O:E ratios of DCD lung use were examined over time using the Cochran-Armitage test.

### Statistical Analysis

Nonparametric Kruskal-Wallis and χ^2^ tests were used to compare donor and recipient characteristics as well as perioperative outcomes for lung transplant recipients. Fisher exact tests were used if sample sizes did not meet the assumptions for χ^2^ testing. The Kaplan-Meier log-rank survival analysis was used to evaluate recipient mortality. As already described, bootstrapping was used to calculate 95% CIs and *P* values for reported O:E ratios. Statistical analyses were performed using RStudio, version 4.2.3 (R Project for Statistical Computing), with statistical significance set as a 2-sided *P* < .05.

## Results

### Study Population and Perioperative Outcomes

**Figure 1.**
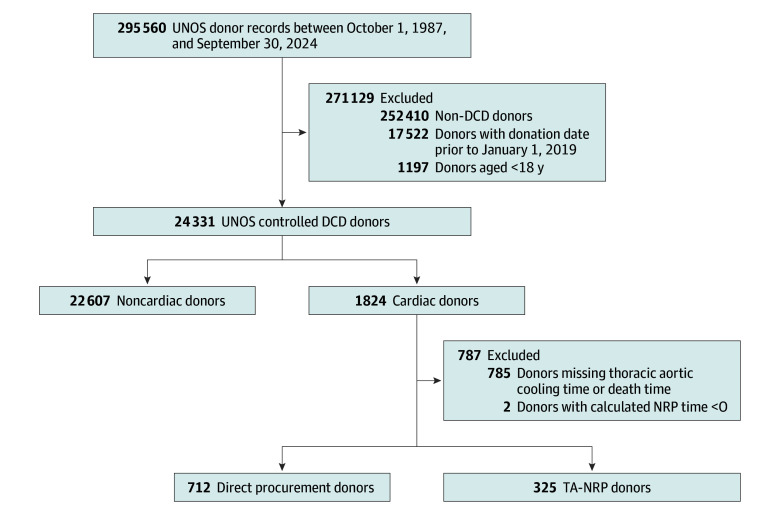
Flowchart of Cohort Selection, Including Inclusion and Exclusion Criteria DCD indicates donation after circulatory death; NRP, normothermic regional perfusion; TA-NRP, thoracoabdominal normothermic regional perfusion; and UNOS, United Network for Organ Sharing.

During the study period, 24 431 controlled DCD donors (8553 [35.0%] female and 15 878 [65.0%] male; median [IQR] age, 49.0 [37.0-58.0] years) met inclusion criteria (Figure 1). Of these donors, 22 607 were classified as noncardiac DCD donors (8232 [36.4%] female and 14 375 [63.6%] male; median [IQR] age, 51.0 [39.0-58.0] years) and 1824 as cardiac DCD donors (321 [17.6%] and 1503 [82.4%] male; median [IQR] age, 32.0 [26.0-38.0] years), with noncardiac DCD donors being significantly older (*P* < .001), less likely to be male (*P* < .001), and more likely to be smokers (6873 [30.4%] vs 227 [12.4%]; *P* < .001) and having lower median (IQR) arterial partial pressure of oxygen to fraction of inspired oxygen (Pao_2_:Fio_2_) ratios (235 [147-343] vs 277 [182-387]; *P* < .001) compared with cardiac DCD donors. Recipients of noncardiac DCD lungs were more likely than cardiac DCD recipients to remain intubated 72 hours after the operation (411 [43.3%] vs 108 [36.9%]; *P* = .050), but both cohorts experienced similar hospital lengths of stay (24.0 [15.0-45.0] days vs 24.0 [15.5-40.0] days; *P* = .58) and rates of postoperative airway dehiscence (17 [1.8%] vs 9 [3.1%]; *P* = .18), respectively (Table 1). The cardiac DCD recipients had better 30-day survival compared with noncardiac DCD (923 of 950 [97.2%] vs 291 of 293 [99.3%]; log-rank *P* = .04) but similar overall survival (28 of 293 [71.9%] vs 8 of 950 [44.4%]; log-rank *P* = .24). Of 1824 hearts recovered from cardiac DCD donors, 1698 (93.1%) were transplanted and 126 (6.9%) were not. Of 126 nontransplanted hearts, 9 were discarded locally, 14 shared and discarded, 16 submitted for research, 10 used for heart valves, and 77 discarded.

**Table 1.** Donor and Recipient Demographics Among Noncardiac DCD and Cardiac DCD Cohorts

Characteristics and outcomes	Patients, No. (%)		*P* value (KW or Fisher exact test)
	Noncardiac DCD	Cardiac DCD	
**Donor characteristics**			
No.	22 607	1824	NA
Age, median (IQR), y	51.0 (39.0-58.0)	32.0 (26.0-38.0)	<.001
Sex			
Female	8232 (36.4)	321 (17.6)	<.001
Male	14 375 (63.6)	1503 (82.4)	
Cause of death			
Anoxia	11 846 (52.4)	891 (48.8)	<.001
CVA or stroke	5029 (22.2)	151 (8.3)	
Head trauma	3809 (16.8)	712 (39.0)	
CNS tumor	38 (0.2)	5 (0.3)	
Other	1885 (8.3)	65 (3.6)	
Smoking history	6873 (30.4)	227 (12.4)	<.001
Missing	627 (3.0)	36 (2.0)	NA
Pao_2_:Fio_2_ ratio, median (IQR)	235 (147-343)	277 (182-387)	<.001
Missing	291 (1.3)	6 (0.3)	NA
Pao_2_:Fio_2_ ratio ≤300	14 819 (65.6)	1013 (55.5)	<.001
Missing	291 (1.3)	6 (0.3)	NA
**Recipient characteristics**			
No.	968	306	NA
Organ received			
Heart-lung	0	7 (2.3)	<.001
Unilateral lung	160 (16.5)	45 (14.7)	
Bilateral lung	808 (83.5)	254 (83.0)	
Ischemic time, median (IQR), h^a^	7.74 (5.97-11.40)	7.85 (5.98-11.80)	.77
Missing	22 (2.3)	13 (4.2)	NA
Distance from donor to recipient center, median (IQR), nautical miles	216 (78-487)	271 (106-624)	.01
**Perioperative outcomes^b^**			
No.	950	293	NA
Acute rejection episode predischarge	125 (13.2)	38 (13.0)	.93
Mortality			
Cumulative	234 (24.6)	36 (12.3)	<.001
30 d	27 (2.8)	2 (0.7)	.04
90 d	61 (6.4)	13 (4.4)	.21
In-hospital	66 (6.9)	12 (4.1)	.07
Missing	8 (0.8)	0	NA
Postoperative intubation at 72 h	411 (43.3)	108 (36.9)	.05
Postoperative airway dehiscence	17 (1.8)	9 (3.1)	.18
Missing	3 (0.3)	1 (0.3)	NA
Postoperative dialysis	121 (12.7)	33 (11.3)	.50
Hospital length of stay, d	24.0 (15.0-45.0)	24.0 (15.5-40.0)	.58
Missing	23 (2.4)	6 (2.0)	
Reintubated	226 (23.8)	78 (26.6)	.27
Missing	4 (0.4)	5 (1.7)	NA
Pao_2_:Fio_2_ ratio			
At 72 h	304 (225-387)	303 (227-381)	.91
Missing	479 (50.4)	144 (49.1)	NA
200-300^c^	136 (14.3)	47 (16.0)	.26
Missing	314 (33.1)	107 (36.5)	NA
<200 or Receiving VA-ECMO	259 (27.3)	64 (21.8)	.12
Missing	314 (33.1)	107 (36.5)	NA
<300 or Receiving VA-ECMO	395 (41.6)	111 (37.9)	.55
Missing	314 (33.1)	107 (36.5)	NA
Postoperative VA-ECMO at 72 h	165 (17.4)	37 (12.6)	.06
Missing	0	1 (0.3)	NA

Abbreviations: CNS, central nervous system; CVA, cerebrovascular accident; DCD, donation after circulatory death; KW, Kruskal-Wallis test; NA, not applicable; Pao_2_:Fio_2_, arterial partial pressure of oxygen to fraction of inspired oxygen ratio; VA-ECMO, venoarterial extracorporeal membrane oxygenation.

^a^ Calculated using available recipient data for donors with 1 or both lungs transplanted: 959 donors had 1 or both lungs transplanted and had lung or heart-lung recipient data available. Of these donors, 933 donated lungs or heart-lung to 1 recipient and 26 to 2 recipients. Thus, 933 + (2 × 26) = 985 recipients were included in these calculations.

^b^ Calculated using available recipient follow-up data: follow-up data were unavailable for 18 noncardiac DCD and 13 cardiac DCD recipients, who were excluded from analysis of outcomes.

^c^ Excluding individuals receiving VA-ECMO.

The cardiac DCD cohort was stratified by procurement technique, with 712 donors undergoing direct procurement and 325 undergoing TA-NRP (Table 2). In total, 787 (43.0%) cardiac DCD donors could not be defined as direct procurement or TA-NRP due to missing time of death or thoracic aorta cooling time. Direct procurement donors (median [IQR] age, 31.0 [26.0-38.0] years) were significantly younger than TA-NRP donors (median [IQR], 33.0 [26.0-40.0] years; *P* = .01); other donor demographics were similar between direct procurement and TA-NRP. However, the TA-NRP cohort had shorter median (IQR) lung ischemic times (6.07 [4.38-9.56] hours vs 8.12 [6.16-12.00] hours; *P* < .001) and distances between the donor and recipient hospital (222 [9-626] nautical miles vs 331 [159-521] nautical miles; *P* = .050). Direct procurement and TA-NRP recipients had similar rates of reintubation and acute rejection episodes, although direct procurement recipients had longer hospital stays. However, TA-NRP recipients’ 90-day mortality rate (0 of 62 vs 9 of 128 patients [7.0%]; *P* = .03) and overall survival (4 of 62 patients [6.5%] vs 21 of 128 [16.4%]; *P* = .04) were significantly better than for direct procurement recipients.

**Table 2.** Donor and Recipient Demographics Among the Direct Procurement and NRP Cohorts

Characteristic	Patients, No. (%)		*P* value (KW or Fisher exact test)
	Direct procurement	TA-NRP	
**Donors**			
No.	712	325	NA
Age, median (IQR), y	31.0 (26.0-38.0)	33.0 (26.0-40.0)	.01
Sex			
Female	143 (20.1)	59 (18.2)	.47
Male	569 (79.9)	266 (81.8)	
Cause of death			
Anoxia	349 (49.0)	140 (43.1)	.14
CVA or stroke	49 (6.9)	32 (9.8)	
Head trauma	291 (40.9)	134 (41.2)	
CNS tumor	3 (0.4)	1 (0.3)	
Other	20 (2.8)	18 (5.5)	
Smoking history	80 (11.2)	44 (13.5)	.25
Missing	11 (1.5)	10 (3.1)	NA
Pao_2_:Fio_2_ ratio, median (IQR)	295 (192-404)	271 (176-388)	.05
Missing	1 (0.1)	1 (0.3)	NA
Pao_2_:Fio_2_ ratio ≤300	368 (51.7)	186 (57.2)	.09
Missing	1 (0.1)	1 (0.3)	NA
Ex vivo lung perfusion			
Left lung	49 (6.9)	15 (4.6)	.15
Missing	421 (59.1)	193 (59.4)	NA
Right lung	47 (6.6)	14 (4.3)	.13
Missing	425 (59.7)	194 (59.7)	NA
**Recipients**			
No.	134	64	NA
Organ received			
Heart-lung	0	5 (7.8)	.007
Unilateral lung	22 (16.4)	9 (14.1)	
Bilateral lung	112 (83.6)	50 (78.1)	
Ischemic time, median (IQR), h^a^	8.12 (6.16-12.00)	6.07 (4.38-9.56)	<.001
Missing	6 (4.5)	2 (3.1)	NA
Distance from donor to recipient center, median (IQR), nautical miles	331 (159-521)	222 (9-626)	.05
**Perioperative outcome^b^**			
No.	128	62	NA
Acute rejection episode predischarge	17 (13.3)	12 (19.4)	.28
Mortality			
Cumulative	21 (16.4)	4 (6.5)	.06
30 d	0	0	NA
90 d	9 (7.0)	0	.03
In-hospital	8 (6.3)	0	.06
Postoperative intubation at 72 h	45 (35.2)	24 (38.7)	.63
Missing	2 (1.6)	1 (1.6)	NA
Postoperative airway dehiscence	5 (3.9)	0	.17
Missing	1 (0.8)	0	
Postoperative dialysis	17 (13.3)	3 (4.8)	.08
Hospital length of stay, d	23.0 (16.0-43.0)	20.0 (13.0-36.0)	.04
Missing	3 (2.3)	1 (1.6)	NA
Reintubated	26 (20.3)	19 (30.6)	.11
Missing	2 (1.6)	1 (1.6)	NA
Pao_2_:Fio_2_ ratio			.83
At 72 h	297 (215-379)	307 (230-393)	
Missing	65 (50.8)	29 (46.8)	NA
200-300^c^	20 (15.6)	11 (17.7)	.80
Missing	47 (36.7)	21 (33.9)	NA
<200 or Receiving VA-ECMO	31 (24.2)	13 (21.0)	.48
Missing	47 (36.7)	21 (33.9)	NA
<300 or Receiving VA-ECMO	51 (39.8)	24 (38.7)	.64
Missing	47 (36.7)	21 (33.9)	NA
Postoperative VA-ECMO at 72 h	18 (14.1)	8 (12.9)	.86
Missing	0	1 (1.6)	NA

Abbreviations: CNS, central nervous system; CVA, cerebrovascular accident; KW, Kruskal-Wallis test; NA, not applicable; Pao_2_:Fio_2_, arterial partial pressure of oxygen to fraction of inspired oxygen ratio; TA-NRP, thoracoabdominal normothermic regional perfusion; VA-ECMO, venoarterial extracorporeal membrane oxygenation.

^a^ Calculated using available recipient data for donors with 1 or both lungs transplanted: 117 donors had 1 or both lungs transplanted and had lung or heart-lung recipient data available. Of these donors, 114 donated lungs or heart-lung to 1 recipient and 3 to 2 recipients. Thus, 114 + (2 × 3) = 120 recipients were included in these calculations.

^b^ Calculated using available recipient follow-up data: follow-up data were unavailable for 6 direct procurement and 2 TA-NRP recipients, who were excluded from analysis of outcomes.

^c^ Excluding donors receiving VA-ECMO.

### Observed and Expected Organ Yields

Among 22 607 noncardiac DCD donors, observed donor lung yield was 988 (4.4%), meaning 4.4% of lungs from noncardiac DCD donors were transplanted. By contrast, cardiac DCD donors had a significantly higher observed yield of 304 donors (16.7%) (*P* < .001). When stratifying the cardiac DCD cohort by procurement technique, there was no significant difference in observed lung yield between cardiac DCD donors who underwent direct procurement vs TA-NRP for heart recovery (133 [18.7%] vs 62 [19.1%]; *P* = .88).

Comparing O:E ratios for lung use revealed that all cohorts exceeded expected lung yields. The noncardiac DCD cohort had an O:E ratio of 1.29 (95% CI, 1.21-1.35; *P* < .001), while the cardiac DCD cohort O:E ratio was 1.79 (95% CI, 1.62-1.96; *P* < .001). Among cardiac DCD donors, TA-NRP donors had the highest O:E ratio (2.00 [95% CI, 1.60-2.43]), and the direct procurement cohort O:E ratio was 1.77 (95% CI, 1.52-1.99). All of these O:E ratios were statistically significant. However, while the cardiac DCD donor O:E ratio was higher than that for noncardiac DCD donors, the O:E ratios did not differ significantly between the TA-NRP and direct procurement groups (*P* = .83).

### Subanalyses

**Figure 2.**
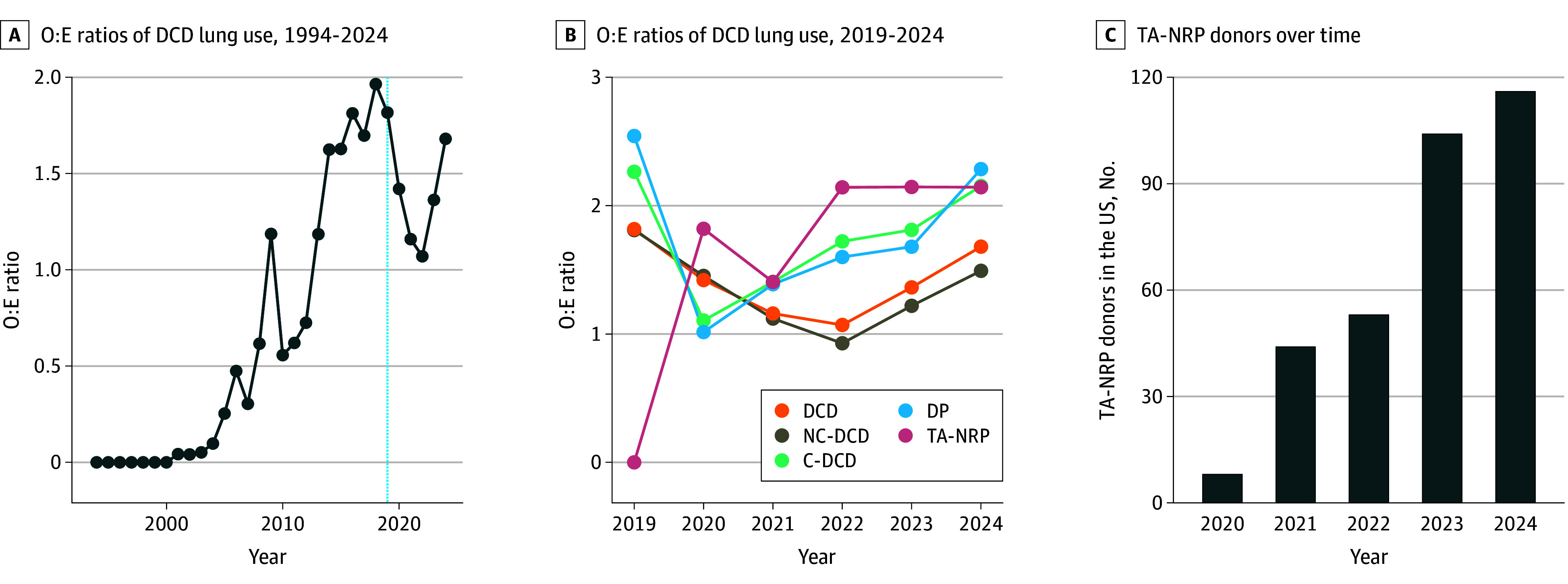
Temporal Trends in Donation After Circulatory Death (DCD) and Thoracoabdominal Normothermic Regional Perfusion (TA-NRP) Lung Use A, Vertical light blue line denotes 2019, the year of the first recorded DCD heart transplant in the US. C-DCD indicates cardiac DCD donor; DP, direct procurement; NC-DCD, noncardiac DCD donor; O:E, observed to expected; and TA-NRP, thoracoabdominal normothermic regional perfusion.

The O:E ratios for donor lung yields were further stratified by year to evaluate temporal trends in lung use. Using all available controlled DCD data (January 1, 1994, through September 30, 2024), the overall DCD (noncardiac DCD plus cardiac DCD) cohort O:E ratios showed significant change over time (Cochran-Armitage test of trend, *P* < .001) (Figure 2A). However, within our study period (January 1, 2019, through September 30, 2024), the DCD O:E ratios lacked a significant temporal trend (Cochran-Armitage test of trend, *P* = .92), despite significantly increased numbers of DCD donors per year (*P* < .001 for trend). Similarly, among the noncardiac DCD, DCD, direct procurement, and TA-NRP (cohorts, O:E ratios remained stable over time despite significant increases in donor counts (*P* < .001 for trend for all cohorts) (Figure 2B).

**Figure 3.**
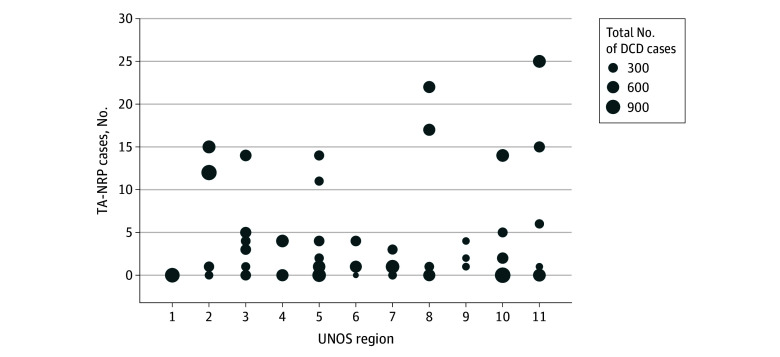
Thoracoabdominal Normothermic Regional Perfusion (TA-NRP) Use by Organ Procurement Organization and United Network for Organ Sharing (UNOS) Region The y-axis reflects total number of TA-NRP cases since January 1, 2019. DCD indicates donation after circulatory death.

Temporal examination of TA-NRP use revealed a steady increase in the number of TA-NRP procurements since 2019 (*P* < .001 for trend) (Figure 2C). We also characterized variations in TA-NRP use among OPOs and UNOS regions. Most OPOs performed fewer than 10 TA-NRP procurements since 2019, while only 5 OPOs performed 20 or more, indicating significant variability in TA-NRP use (Figure 3).

## Discussion

In this cohort study involving analyses of the UNOS registry, we used an SRTR risk-adjusted model to assess the association of simultaneous heart procurement with DCD lung use. We found that simultaneous heart procurement was associated with increased use of lungs recovered from DCD donors. Additionally, when comparing 2 distinct techniques for cardiac DCD, we found that TA-NRP and direct procurement were associated with similar rates of lung use with DCD. To our knowledge, this is the largest study evaluating the association of procurement techniques with lung use from DCD donors and the only study to develop risk-adjusted O:E ratios for this purpose. Furthermore, we observed improved survival among recipients after donor TA-NRP, contrasting with previous smaller studies showing similar survival between TA-NRP and direct procurement. Our results suggest that using TA-NRP to recover DCD hearts is not negatively associated with lung use and may, in fact, be associated with improved posttransplant survival.

TA-NRP has gained traction in recent years as a method to reanimate hearts received through DCD and assess their viability for transplantation, demonstrating improved DCD liver and kidney transplantation outcomes.^15,16^ Increased DCD liver use has also been demonstrated in recipients from donors with TA-NRP compared with donors undergoing direct procurement.^17^ Nevertheless, concerns persist that it may damage otherwise healthy lungs and negatively impact DCD lung use. Rapid access to the thoracic cavity and prolonged regional mechanical circulatory support can precipitate extensive blood loss and necessitate transfusions, while left ventricular dilation or increased left atrial pressures with VA-ECMO can cause pulmonary edema—both well-documented potential sources of injury.^3^ Despite these concerns, our analysis found that both direct procurement and TA-NRP cohorts had better-than-expected DCD lung use (O:E 1.77 vs 2.00; *P* < .001 for both). Notably, the 2 cohorts did not differ significantly, indicating both methods are reasonable options for heart recovery from a lung use perspective.

Previous reports have cited raw percentages of lung use rates but have been limited by small cohorts and have not adjusted for donor characteristics that directly correlate with use.^4,8,9^ Simply comparing actual lung use between cohorts is not entirely meaningful, as it is unlikely that all donors were ideal lung donation candidates. The probability of successful organ yield varies across both donors and different organs within the same donor. Our analysis provides a detailed overview of lung yield in DCD donors by leveraging SRTR data to adjust for donor factors, allowing for comparison of observed DCD lung yields against reasonable standards, potentially alleviating previous concerns about whether TA-NRP interferes with lung recovery. Additionally, we demonstrated a substantial yearly increase in use of the TA-NRP technique (*P* < .001 for trend) but stable O:E ratios over time (Cochran-Armitage test for trend, *P* = .24), further supporting the argument that TA-NRP does not negatively impact donor lung yield as experience has accumulated.

Despite a TA-NRP O:E ratio that was greater than expected and comparable to the direct procurement group, anecdotal experiences from transplant teams still reveal concern about the potential of TA-NRP to damage lung allografts. These concerns may be partially reflected in variable OPO experience with TA-NRP. Our analysis found heterogeneity in OPO TA-NRP volumes: only 5 OPOs performed 20 or more TA-NRP procurements since 2019, while over half of OPOs performed under 10 in the same period. Although O:E ratios were similar between TA-NRP and direct procurement cohorts, variation may exist in TA-NRP O:E ratios at the OPO level. As low-volume OPOs increase TA-NRP volume and gain experience with the technique, DCD lung use may improve, potentially resulting in significantly higher O:E ratios nationally. Based on our analysis, anecdotal cases of poor lung quality following TA-NRP procurement should not deter transplant teams and OPOs from the technique. Instead, standardizing implementation of this technique should be prioritized, especially given the variations that currently exist.^3,18,19^ In fact, the argument to use TA-NRP extends beyond lung use. A study by Bakhtiyar et al^20^ demonstrated that TA-NRP is significantly cheaper and generates greater organ yield per donor compared with direct procurement followed by ex situ machine perfusion. Additionally, while perioperative outcomes were not the primary focus of our study, we also observed improved 90-day and overall survival in the TA-NRP cohort vs direct procurement, contrasting with prior work that has demonstrated comparable outcomes between the groups.^4,8,9^ The discrepancy likely stems from the larger sample size and more comprehensive analysis in our study. Further in-depth assessment of allograft and patient survival should be prioritized as case volume and experience accumulate. As prior analyses have shown a positive correlation between transplant center experience and posttransplant outcomes with ex vivo lung perfusion, future research should evaluate whether a similar association exists for TA-NRP.^21^

### Limitations

There are several limitations in our study. First, the UNOS SRTR registry does not explicitly track TA-NRP use.^4,8,22^ Consequently, we inferred use of TA-NRP from time elapsed between death and thoracic organ cooling. While we believe that a minimum threshold of 15 minutes between death and thoracic aorta cold flush most accurately captures TA-NRP donors, and this has been previously validated, there remains heterogeneity and a lack of consensus regarding the minimum threshold. Additionally, 43% of our 1824 cardiac donors were missing either time of death or thoracic aortic cooling time, necessitating exclusion and potentially introducing bias. The lack of a discrete field for these important data, compounded by missingness in other fields, highlights the importance of the OPTN modernization effort, especially with regard to data sciences. As TA-NRP, machine perfusion, and other technological advances become increasingly embedded in the national landscape of solid organ transplantation, the OPTN should make explicit provisions to collect important data for these donors to facilitate investigation of the impact of these changes on organ use and outcomes. These changes will potentially provide additional data to address the ethical concerns associated with NRP use as well.^23^ Furthermore, differences in lung use and outcomes between TA-NRP and abdominal-NRP remain unclear; improved data collection could help answer this question. Last, our use of the SRTR risk-adjustment model for donor yield presents a potential limitation. These models are trained on recent data and updated biannually, meaning that the 2024 model we used was based on data from the second half of 2023.^10^ Thus, our risk-adjusted analysis is based on current practices and trends, in essence using today’s expectations to evaluate yesterday’s donors, potentially underestimating the O:E ratios we developed. However, given that our observed yields exceeded expected yields, we believe this only strengthens our conclusion that TA-NRP does not impede lung use and may, in fact, be associated with improved recovery.

## Conclusions

In this national cohort study of DCD donors, we used risk-adjusted models from the SRTR to evaluate the association of simultaneous heart procurement, including direct procurement and TA-NRP techniques, with use of DCD donor lungs. We found that simultaneous heart procurement was not adversely associated with DCD lung use, nor was use of TA-NRP for cardiac DCD associated with decreased use of lung allografts. Importantly, we identified significant heterogeneity in TA-NRP use and experience among OPOs, which may lead to varying rates of lung use when using TA-NRP. Our findings suggest that OPOs and transplant teams should not be deterred from pursuing cardiac DCD transplantation, regardless of procurement technique employed. Additionally, OPOs, transplant teams, and the OPTN should strive to standardize procurement techniques, especially for multiorgan DCD, to optimize all DCD organ yields. As UNOS data reporting improves and NRP experience accumulates in the US, future studies should evaluate the impact of ex vivo lung perfusion and abdominal NRP vs TA-NRP on lung use, as well as potential relationships between center volume of TA-NRP cases and clinical posttransplant outcomes.

## Notes

Supplement.
Data Sharing Statement
