# Supplementary material for: Thoracoabdominal Normothermic Regional Perfusion and Donation After Circulatory Death Lung Use
Source: JAMA Netw Open. 2025 Feb 17;8(2):e2460033. doi: 10.1001/jamanetworkopen.2024.60033 (PMC11833517; doi:10.1001/jamanetworkopen.2024.60033)
Supplement: Supplement. — Data Sharing Statement [file jamanetwopen-e2460033-s001.pdf]

Data Sharing Statement

Alderete. Thoracoabdominal Normothermic Regional Perfusion and Donation After Circulatory Death Lung Use. *JAMA Netw Open*. Published February 17, 2025.
doi:10.1001/jamanetworkopen.2024.60033

Data
Data available: No

Additional Information
Explanation for why data not available: Due to HRSA And agreements with UNOS. Raw R coding can be providing however.
